# Identification of patients with myocarditis by cardiovascular magnetic resonance: T1- and T2-mapping are superior compared to conventional semi-quantitative techniques

**DOI:** 10.1186/1532-429X-16-S1-P287

**Published:** 2014-01-16

**Authors:** Ulf K Radunski, Sebastian Bohnen, Gunnar Lund, Christian Stehning, Katharina Koopmann, Charlotte Rauwald, Lukas Radziwolek, Gerhard Adam, Stefan Blankenberg, Kai Muellerleile

**Affiliations:** 1 Cardiology, University Heart Center, Hamburg, Germany; 2 Radiology, University Medical Center, Hamburg, Germany; 3 Philips Research, Hamburg, Germany

## Background

Current semi-quantitative cardiovascular magnetic resonance (CMR) techniques are of limited diagnostic value in patients with suspected myocarditis. T1- and T2-mapping CMR are promising novel quantitative approaches to assess myocardial injury. This study evaluated the performance of T1 and T2 mapping CMR to identify patients with myocarditis in comparison with conventional CMR techniques.

## Methods

This study included 104 patients with myocarditis and 26 controls, who underwent CMR at 1.5 Tesla. The CMR protocol included conventional sequences to assess myocardial edema (T2w-STIR), Early- (T1w-TSE) and Late-Gadolinium-Enhancement (PSIR). T1 quantification was performed using the modified Look-Locker inversion-recovery (MOLLI) sequence before and 15 minutes after administration of 0.075 mmol/kg Gadolinium-BOPTA. T2 quantification was performed using a free-breathing, navigator-gated multi-echo sequence. T1, T2 and extracellular volume (ECV) maps were calculated with a dedicated plug-in written for the OsiriX software. The diagnostic performance was compared between conventional parameters and global myocardial T1 (native and post-contrast), ECV and T2.

## Results

The ROC areas-under-the-curve (AUC) to discriminate between patients with myocarditis and controls were 0.58 (p = 0.19) for the signal-intensity ratio myocardium/skeletal muscle on T2w-STIR, 0.67 (p = 0.01) for early myocardial enhancement on T1w-TSE and 0.80 (p < 0.0001) for presence of Late-Gadolinium-Enhancement, respectively. Furthermore, the AUC to discriminate between patients with myocarditis and controls were 0.77 (p < 0.0001) for native global myocardial T1, 0.59 (p = 0.14) for post-contrast global myocardial T1, 0.85 (p < 0.0001) for global myocardial ECV and 0.79 (p = 0.0001) for global myocardial T2, respectively.

## Conclusions

T1 and T2 mapping provide a superior performance to identify patients with myocarditis compared to conventional CMR techniques. In particular, the estimation of global myocardial ECV by T1 mapping improves the diagnostic value of CMR in patients with clinically suspected myocarditis.

## Funding

Marija-Orlovic-Foundation.

